# Grape seed extract prevents skeletal muscle wasting in interleukin 10 knockout mice

**DOI:** 10.1186/1472-6882-14-162

**Published:** 2014-05-20

**Authors:** Bo Wang, Guan Yang, Xingwei Liang, Meijun Zhu, Min Du

**Affiliations:** 1 Department of Animal Sciences, Washington State University, Pullman, WA 99163, USA; 2 School of Food Science, Washington State University, Pullman, WA 99164, USA

**Keywords:** Apoptosis, Atrophy, Grape seed extract, IL-10, Inflammasome, Inflammation, Skeletal muscle, Wasting

## Abstract

**Background:**

Muscle wasting is frequently a result of cancers, AIDS, chronic diseases and aging, which often links to muscle inflammation. Although grape seed extract (GSE) has been widely used as a human dietary supplement for health promotion and disease prevention primarily due to its anti-oxidative and anti-inflammative effects, it is unknown whether GSE affects muscle wasting. The objective is to test the effects of GSE supplementation on inflammation and muscle wasting in interleukin (IL)-10 knockout mice, a recently developed model for human frailty.

**Methods:**

Male IL-10 knockout (IL10KO) C57BL/6 mice at 6 weeks of age were assigned to either 0% or 0.1% GSE (in drinking water) groups (n = 10) for 12 weeks, when skeletal muscle was sampled for analyses. Wild-type C57BL/6 male mice were used as controls.

**Results:**

*Tibialis anterior* muscle weight and fiber size of IL10KO mice were much lower than wild-type mice. IL10KO enhanced nuclear factor kappa-light-chain-enhancer of activated B cells (NF-κB) signaling and inflammasome formation when compared to wild-type mice. Phosphorylation of anabolic signaling was inhibited, whereas muscle specific ubiquitin ligase, AMP-activated protein kinase (AMPK) and apoptotic signaling were up-regulated in IL10KO mice. GSE supplementation effectively rectified these adverse changes in IL10KO muscle, which provide an explanation for the enhanced muscle mass, reduced protein degradation and apoptosis in GSE supplemented mice compared to IL10KO mice without supplementation.

**Conclusion:**

GSE supplementation effectively prevents muscle wasting in IL10KO mice, showing that GSE can be used as an auxiliary treatment for muscle loss associated with chronic inflammation and frailty.

## Background

Muscle wasting is frequently a consequence of cancers, AIDS, immobilization and fasting [1]. During ageing, there is a gradual loss of muscle mass and a diminished capacity to reverse that loss, resulting in weakness and frailty [2,3]. Currently, there are few options to prevent or slow down muscle wasting and, thus, there are compelling reasons to develop new medicines or nutritional remedies that can maintain skeletal muscle mass [3].

Muscle wasting is frequently associated with chronic inflammation [4,5]. Polyphenolic compounds are known for their anti-oxidative and anti-inflammatory effects, and have preventive or therapeutic effects on a number of metabolic diseases including obesity, diabetes, hypercholesterolemia, cardiovascular diseases and cancer [6-12]. Resveratrol, the best studied polyphenol, improves mitochondrial function, muscle strength and endurance capacity by activating silent mating type information regulation 2 homolog 1 (SIRT1) and AMP-activated protein kinase (AMPK) [13,14]. However, up to now, the role of polyphenolic compounds in inflammation and muscle wasting has not been defined. Grape seed extract (GSE) is a by-product of the winery and grape juice industry, which is rich in polyphenolic compounds [15]. Consistently, GSE is known for its anti-oxidative and anti-inflammatory effects [16,17], and alleviates oxidative stress in skeletal muscle [18], which prompted us to examine the role of GSE in preventing muscle wasting.

Interleukin 10 knockout (IL10KO) mice is a recently proposed model for studying low-grade inflammation, multisystemic decline and frailty [19]. IL10KO mice show accelerated muscle loss and weakness [20], and also chronic inflammation, ideal for assessing inflammation associated muscle wasting and frailty [19,21]. Using this mouse model, the objective of this study is to test the effectiveness of GSE in preventing muscle loss in IL10KO mice and further explore underlying mechanisms.

## Methods

### Animals and diets

All animal procedures were approved by the Washington State University Animal Care and Use Committee. Wild-type (WT) C57BL/6 and homozygous IL-10 deficient mice (B6.129P2-Il10^tm1Cgn^/) were initially purchased from Jackson Lab (Bar Harbor, ME, USA) and then bred under pathogen-free (SPF) conditions in the Experimental Animal Laboratory Building at Washington State University. Mice had free access to food (a standard rodent diet) and drinking water. IL10KO female mice at 6 weeks of age were randomly assigned into 2 groups (n = 10 for each group), receiving either 0 or 0.1% GSE (g/ml in drinking water, equal to ~0.2 mg/g body weight/day) for 12 weeks; WT female mice aged 6 weeks were used as controls. Water was changed daily to avoid the possible oxidation of functional compounds in GSE. There was no difference for the amount of water and diet consumed among these groups. Similar dosages of GSE have been used in previous studies [22,23]. GSE used in this study is a commercial GSE product (Gravinol-S) purchased from OptiPure Chemco Industries Inc. (Los Angeles, CA). Per company product specification sheet, it contains a minimum 95% flavonols, of which 82% are oligomeric proanthocyanidins (OPCs), and 12% being the highly active monomeric OPCs. The composition of GSE was further analyzed by mass spectrometry in our lab and the major components include catechin monomer 7.3%, dimer 35.8%, trimer 38.6%, tetramer 12.8%, pentamer 5.4%, and trace amount of hexamer.

### Sampling

Mice were anaesthetized by fluorine inhalation before euthanization by cervical dislocation. Intact *Tibialis anterior* was isolated from hind legs, weighed before fixing for paraffin embedding. *Gastrocnemius* muscle was isolated and frozen in liquid nitrogen and then stored under -80°C until analyses.

### Antibodies and chemicals

Antibodies against nuclear factor kappa-light-chain-enhancer of activated B cells (NFκB) p65 (#4764), phospho-p65 (#3033), Akt (4691), phospho-Akt (#Ser473), AMPKα (#6707), phospho-AMPKα (#4188), mammalian target of rapamycin (mTOR) (#2983), phospho-mTOR (#5536) were purchased from Cell Signaling (Danvers, MA). NACHT, LRR and PYD domains-containing protein 3 (NLRP3) antibody (PA1665) was purchased from Boster Biological Technology (Fremont, CA). IRDye 800CW goat anti-rabbit secondary antibody and IRDye 680 goat anti-mouse secondary antibody were bought from LI-COR Biosciences (Lincoln, NE). Caspase-1 Fluorometric Assay Kit (#K110-100) was purchased from Bio Vision (Milpitas, CA). Apoptosis Kit TACS® XL DAB (diaminobenzidine) Kit (#4810-60-K) was purchased from R&D system (Minneapolis, MN).

### Immunoblotting analysis

Immunoblotting analyses were conducted according to the procedures previously described [24]. Membranes were visualized by Odyssey infrared imaging system (LI-COR Biosciences). Density of bands was quantified and then normalized according to the β-tubulin content.

### Quantitative real time PCR

Total mRNA was extracted from *Gastrocenemius* muscle using Trizol reagent (Invitrogen, Carlsbad, CA), treated with deoxyribonuclease, and reverse transcribed into cDNA using an iScript cDNA synthesis kit (Bio-Rad, Hercules, CA). Real time-PCR was performed on a CFX ConnectTM Real-Time PCR detection system (Bio-Rad) using SYBR Green RT-PCR kit from Bio-Rad. The following cycle parameters were used: 34 three-step cycles of 95°C, 20 sec; 55°C, 20 sec; and 72°C, 20 sec. Primer sequences and their respective PCR fragment lengths were as follows: IL-1β (77 bp), forward 5′- TCGCTCAGGGTCACAAGAAA-3′ and reverse 5′-CATCAGAGGCAAGGAGGAAAAC-3′ ; IL-18 (89 bp), forward 5′- ATGCTTTCTGGACTCCTGCCTGCT-3′ and reverse 5′- GGCGGCTTTCTTTGTCCTGATGCT-3′; tumor necrosis factor (TNF)α (67 bp), forward 5′- TGGGACAGTGACCTGGACTGT-3′ and reverse 5′- TTCGGAAAGCCCATTTGAGT-3′ ; 18S (110 bp) forward 5′-TGCTGTCCCTGTATGCCTCT-3′, and reverse 5′-TGTAGCCACGCTCGGTCA-3′. After amplification, a melting curve (0.01°C/sec) was used to confirm product purity, and agarose gel electrophoresis was performed to confirm that only a single product of the right size was amplified. Relative mRNA content was normalized to 18S rRNA content.

### Histochemical staining and image analysis

Muscle tissue sections (5 μm) were deparaffinized, rehydrated, and used for Masson’s trichrome staining [25], which stains muscle fibers red, nuclei black, and collagen blue. Muscle fiber sizes were measured using the ImageJ software (National Institute of Health, Baltimore, MD) and at least 400 muscle fibers per animal were measured (8 images per section and 5 sections at 50 μm interval per mice). To measure the apoptotic level of skeletal muscle cells, 8 images per section and 2 sections per mice were stained by Apoptosis Kit. Normal cells were stained blue and apoptotic cells were black. All images were analyzed at 200 × magnification.

### Statistical analysis

All data were analyzed using the GLM procedure of SAS (SAS Inst. Inc., Cary, NC), pairwise comparison was performed using fisher’s LSD procedure. Arcsine transformation was applied on percentage data before analysis. Mean values and standard errors of the mean were reported. *P* < 0.05 was considered significant.

## Results and discussion

**Figure 1:**
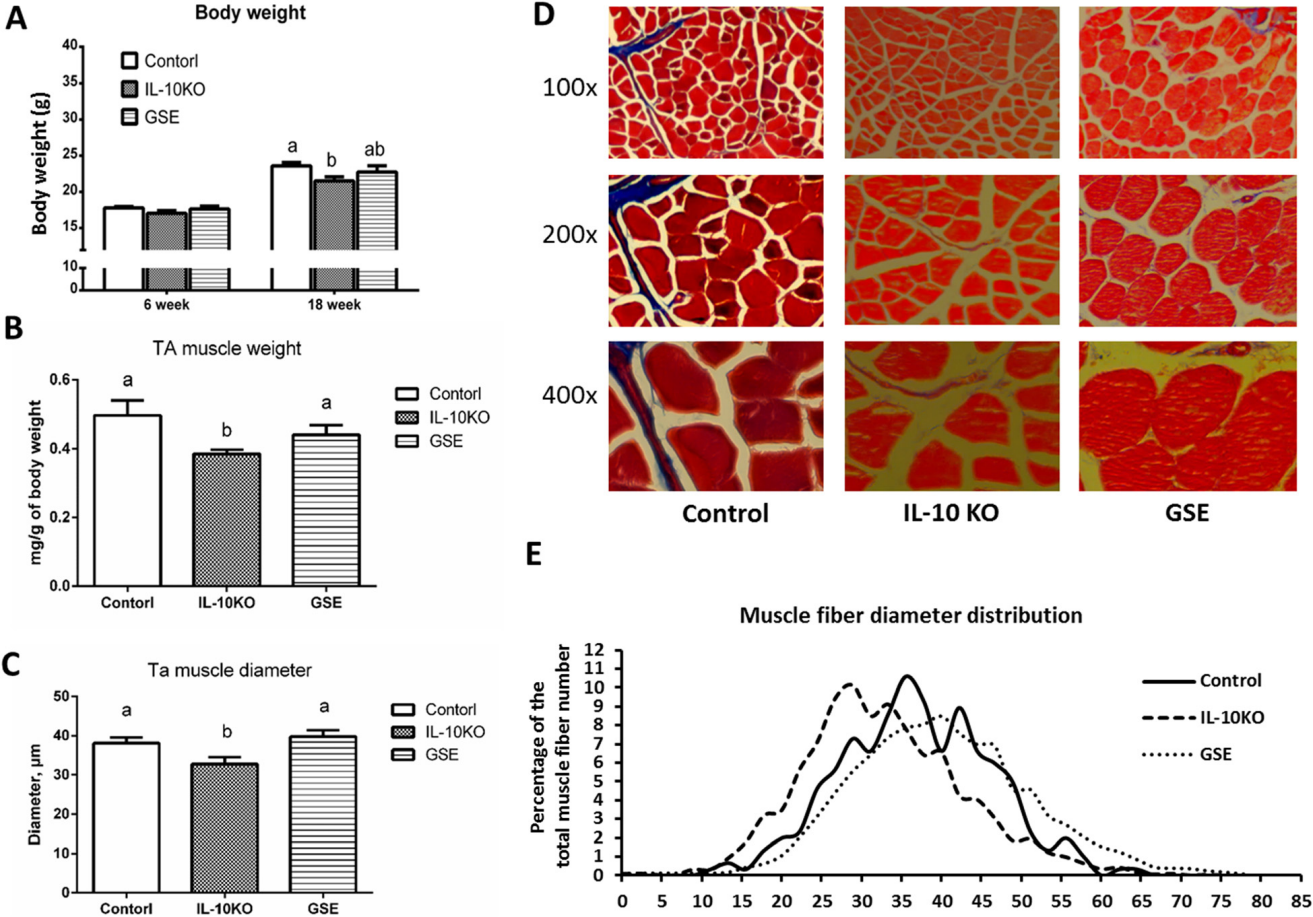
**GSE prevented the *Tibialis anterior *muscle weight loss and the reduction in muscle fiber diameters in IL10 knockout mice. (A)** Body weight of mice aged 6 weeks and 18 weeks. **(B)***Tibialis anterior* muscle weight. **(C)** Average muscle fiber diameter. **(D)** Trichrome staining of muscle. **(E)** Muscle fiber size distribution. (Bars with different letters differ significantly, *P* < 0.05; n = 10; mean ± SE).

IL10KO reduced weight gain when compared to WT mice, and GSE supplementation improved weight gain of IL10KO (Figure 1A). The *Tibialis anterior* muscle weight of IL10KO mice was lower than that of control mice, while GSE supplementation attenuated muscle loss in IL10KO mice (Figure 1B). We further compared the muscle structure among these treatments. As shown by Trichrome staining, IL10KO mice had smaller average fiber diameter (Figure 1C) and more abundant small muscle fibers (Figure 1E). However, the muscle fiber size distribution of GSE treated mice was almost the same as control mice and no difference in average fiber size was detected between these two groups. Microscopically, the muscle fibers in GSE treated mice and control mice were round and larger than those of IL10KO mice without supplementation (Figure 1D). These data clearly show that GSE, despite a low dose, was effective in preventing muscle loss in IL10KO mice. These data are consistent with a study showing that epigallocatechin-3-gallate, a major polyphenolic component in green tea, was effective in preventing cancer cachexia in mice [26].

**Figure 2:**
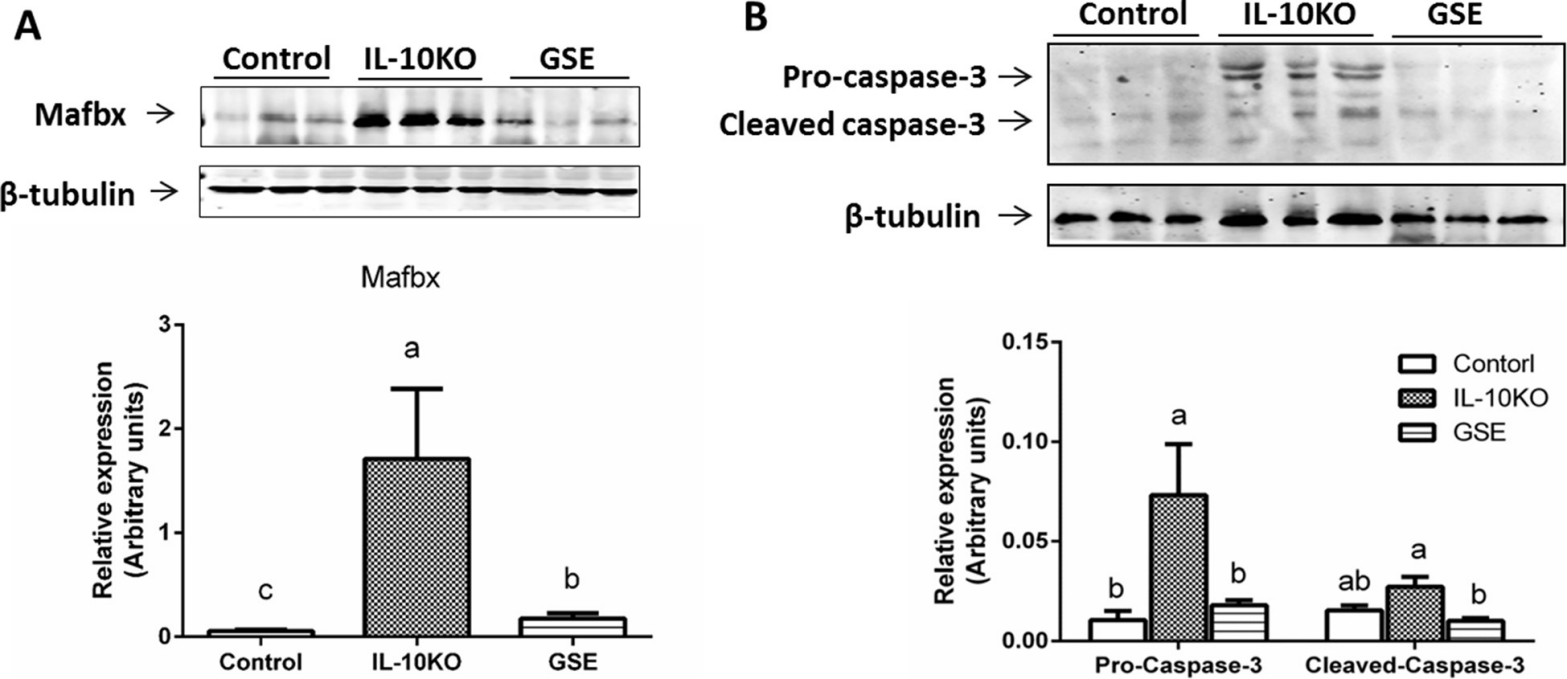
**GSE reduced ubiquitin ligase and caspase-3 expression in IL10 knockout mice. (A)** Atrogin-1/Mafbx content by immunoblotting. **(B)** Pro-caspase 3 and activated caspase 3 contents by immunoblotting. (Bars with different letters differ significantly, *P* < 0.05; n = 10; mean ± SE).

**Figure 3:**
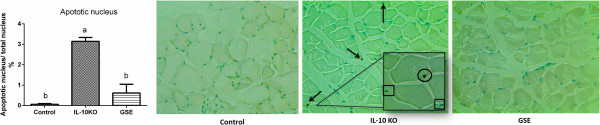
**GSE reduced apoptotic nuclear number in IL10 knockout mice.***In situ* staining of nuclei undergoing apoptosis (Arrows and circles point to nuclei stained black, which were undergoing apoptosis, and boxes point to nuclei stained blue, which were normal). (Bars with different letters differ significantly, *P* < 0.05; n = 10; mean ± SE).

Both ubiquitin –proteasome pathway and apoptosis contribute to skeletal muscle wasting with age [27]. Muscle-specific ubiquitin ligases, muscle atrophy F box (MAFbx) and muscle RING finger 1 (MuRF1), are crucial regulators of myofibrillar protein breakdown [28]. To figure out how GSE prevented muscle wasting in IL10KO mice, the protein content of atrogin-1/MAFbx was measured. As expected, GSE supplementation reduced atrogin-1/MAFbx content in IL10KO to a level identical with WT mice (Figure 2A). In addition to protein degradation, apoptosis leads to the loss of muscle fibers and myogenic cells. Therefore, the activation of caspase 3, a primary executing caspase, was further analyzed. The content of pro-caspase 3 and activated-caspase 3 were dramatically increased in IL10KO mice, GSE supplementation reduced caspase 3 content (Figure 2B). Furthermore, 3.2% of nuclei underwent apoptosis in IL10KO mice, but apoptotic nuclei were hardly detectable in either GSE treated or WT mice (Figure 3). Aggregated, these data show that GSE supplementation strongly counteracted apoptosis and protein degradation in skeletal muscle of IL10KO mice.

**Figure 4:**
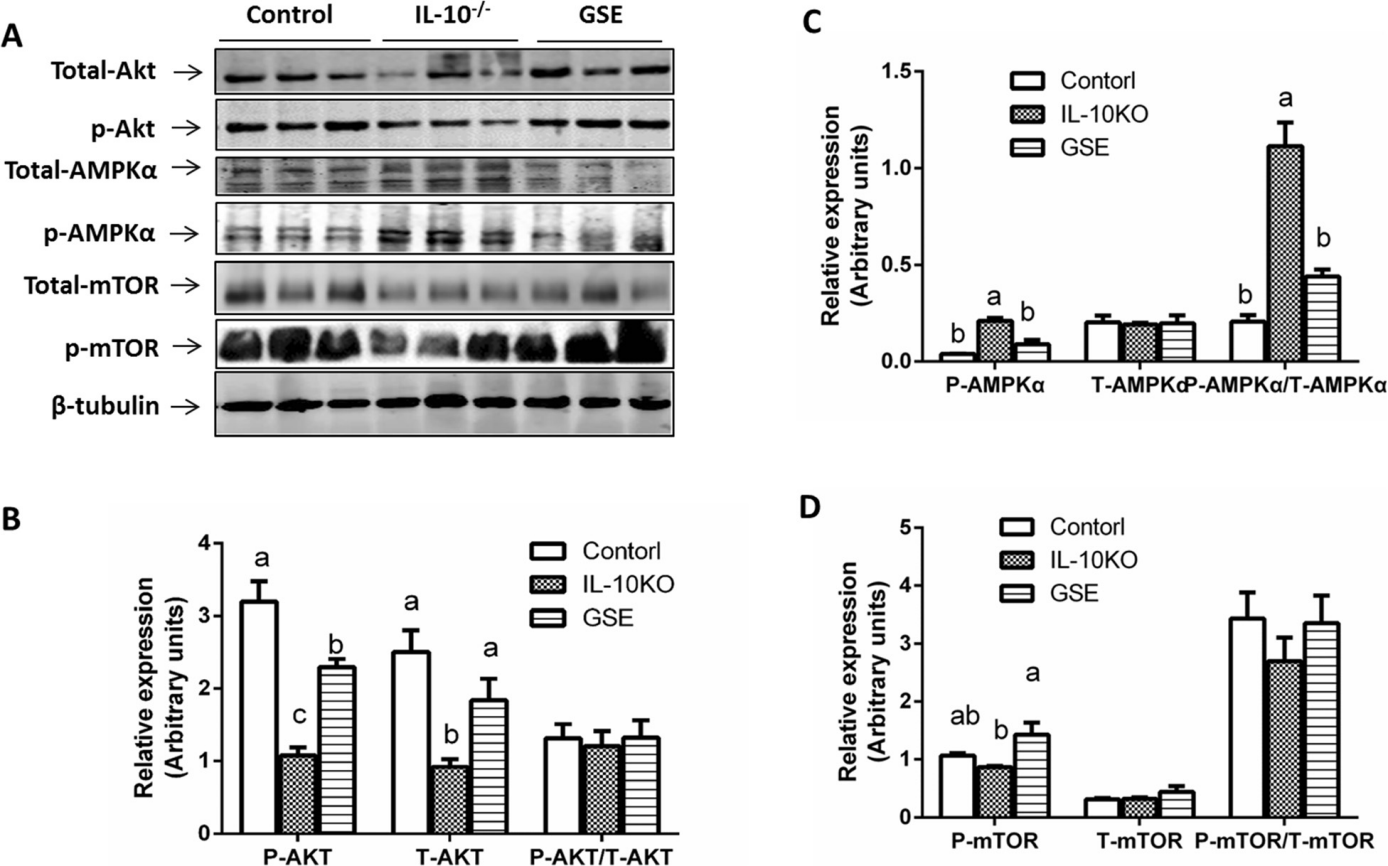
**GSE activated Akt, mTOR and AMPK signaling in IL10 knockout mice. (A)** Representative immunoblots of total Akt, AMPKα, mTOR and their phosphorylated forms. **(B)** Quantitative data of total and phosphorylated Akt **(C)** Quantitative data of total and phosphorylated AMPKα **(D)** Quantitative data of total and phosphorylated mTOR (Bars with different letters differ significantly, *P* < 0.05; n = 10; mean ± SE).

Protein kinase B (Akt) signaling negatively regulates atrogin-1/MAFbx expression and apoptosis [28-30]. To explore mechanisms associated with the down-regulation of protein degradation and apoptosis, we analyzed the phosphorylation of Akt and mTOR. Excitingly, the phosphorylation of Akt and mTOR was enhanced in GSE mice (Figure 4A). As a major growth promoting signaling pathway, the activation of Akt in the muscle of GSE mice provides an explanation for the increased muscle mass in IL10KO mice. We further analyzed AMPK, because it had been reported that resveratrol activates AMPK and improves mitochondria function of skeletal muscle [13,31]. However, we found that AMPKα phosphorylation was elevated in IL10KO mice (Figure 4A), whereas GSE supplementation prevented AMPKα phosphorylation in IL10KO mice. We had been expecting the opposite. Nevertheless, these data are consistent with the observation in aging people, where AMPK basal activity was enhanced [32], due to compromised cellular energetics [33]. Thus, GSE inhibits AMPK activity through enhancing mitochondrial function and cellular energetics in muscle, similar to resveratrol [34].

**Figure 5:**
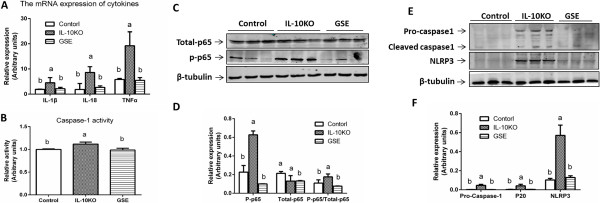
**GSE reduced inflammation and inflammasome activation in IL10 knockout mice. (A)** mRNA expression of inflammatory cytokines. **(B)** Caspase 1 activity **(C, D)** NF-κB p65 phosphorylation by immunoblotting. **(E, F)** Pro-caspase 1 and activated caspase 1 contents by immunoblotting. (*P < 0.05; n = 10; mean ± SE). (Bars with different letters differ significantly, *P* < 0.05; n = 10; mean ± SE).

Chronic inflammation is known to inhibit Akt signaling and induce muscle wasting [35]. As a chronic inflammation model, IL10KO mice suffer from mild inflammation due to IL-10 deficiency. Akt activation and reduced muscle wasting in GSE mice is likely due to the anti-inflammatory effects of GSE. To check whether the protective effects of GSE on IL10KO mice were via its anti-inflammatory effects, we analyzed the expression of inflammatory cytokines. As expected, IL10KO mice had a high level mRNA expression of IL-18, IL-1β and TNFα when compared to GSE supplemented and control mice (Figure 5A). In addition, the phosphorylation of p65, a key mediator of inflammatory NF-κB signaling, was also reduced by GSE in IL10KO mice (Figure 5C), showing the down-regulation of inflammation.

The maturation and secretion of IL-1β and IL-18 are tightly regulated by a diverse class of cytosolic complexes known as the inflammasome, which is associated with inflammation [36]. Upon activation, NLRP3 aggregates with cytosolic oligomers with apoptosis-associated speck-like protein (ASC) to form inflammasome [37], which then triggers activation of caspase-1. Caspase-1, in turn cleaves pro-IL-1β and pro-IL-18 to produce mature IL-1β, and IL-18 [38]. Here, we found that GSE reduced the contents of NLRP3, pro-caspase-1 and cleaved caspase-1 in IL10KO mice (Figure 5E); consistently, the activity of caspase-1 was also reduced in GSE muscle (Figure 5B). Therefore, GSE inhibited inflammation and the activation of inflammasome in the skeletal muscle of IL10KO mice, which is likely associated with the anti-oxidative capacity of GSE because reactive oxygen species induces the activation of NLRP3 inflammasome and inflammation [39].

## Conclusions

**Figure 6:**
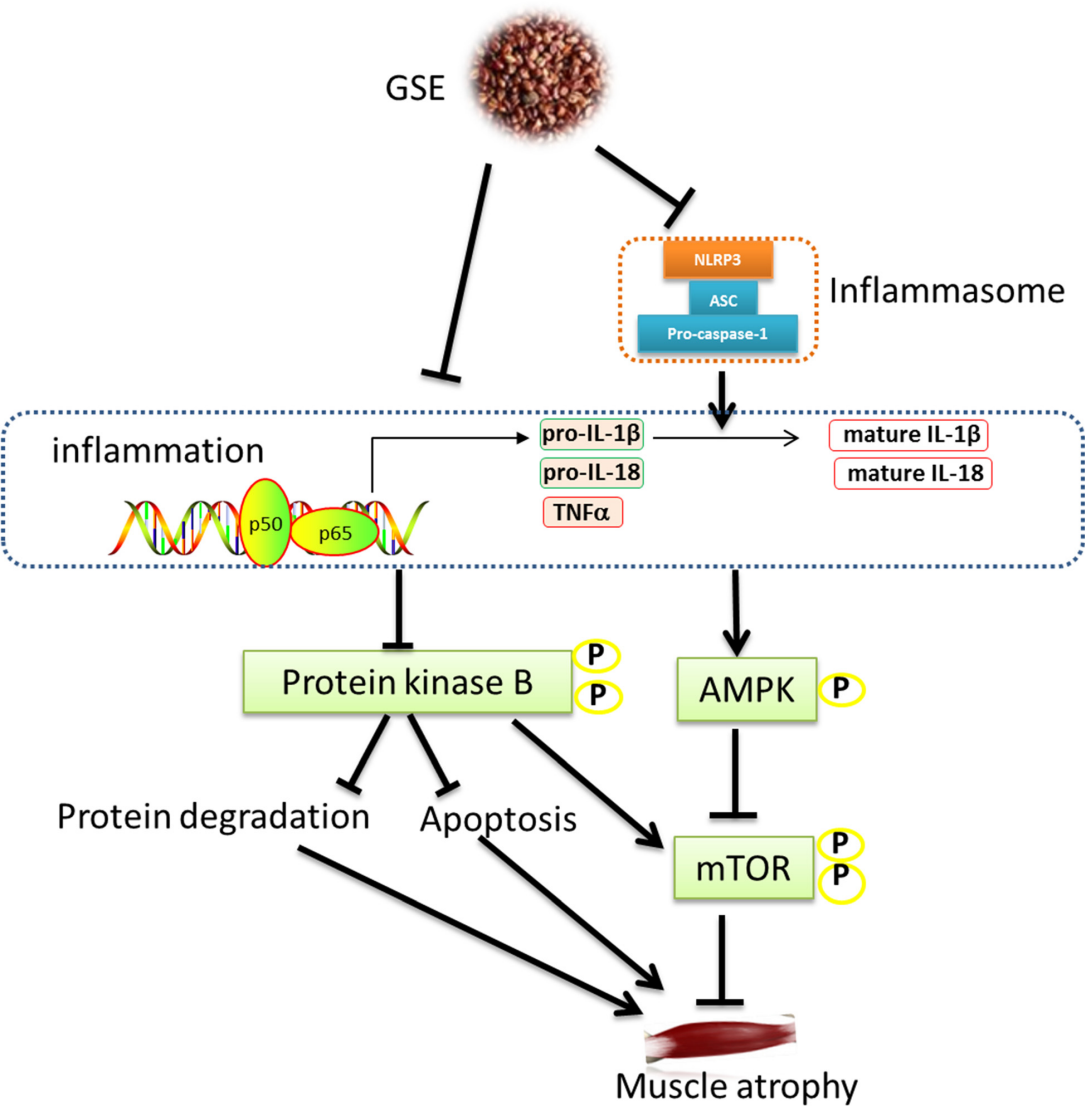
Mechanisms protecting muscle wasting due to GSE supplementation in IL10 knockout mice.

In summary, to the knowledge of authors, for the first time, we found that GSE supplementation prevents muscle loss in a muscle frailty model. The beneficial effects of GSE on muscle loss are likely derived from the down-regulation of chronic inflammation, which reduces protein degradation and apoptosis (Figure 6). Therefore, GSE administration may be used as an auxiliary therapeutic treatment or preventive strategy for inflammation related muscle wasting and frailty.

## Abbreviations

Akt: Protein kinase B; ASC: Apoptosis-associated speck-like protein; GSE: Grape seed extract; IL-10: Interleukin 10; KO: Knockout; MAFbx: Muscle atrophy F box; NF-κB: Nuclear factor kappa-light-chain-enhancer of activated B cells; TNFα: Tumor necrosis factor α; AMPKα: AMP-activated protein kinase α; mTOR: The mechanistic target of rapamycin.

## Competing interests

All authors are in agreement with the content of the manuscript and declare no financial or intellectual conflicts of interests regarding this study.

## Authors’ contributions

M. Du and M. J. Zhu conceived the study. M. Du, M. J. Zhu and Bo Wang designed the trial. Guan Yang performed animal feeding and management. Bo Wang, Guan Yang and Xingwei Liang sacrificed the mice and collected samples. Bo Wang performed sample analysis and data analysis. Bo Wang and M. Du wrote and revised the manuscript. All authors read and approved the final manuscript.

## Pre-publication history

The pre-publication history for this paper can be accessed here:

http://www.biomedcentral.com/1472-6882/14/162/prepub
